# Laparoscopic and Other Intrafascial Hysterectomy Techniques or Mucosal Ablation—A Choice for Maximum Organ Conservation

**DOI:** 10.1155/DTE.2.61

**Published:** 1995

**Authors:** Kurt Semm, Enrique Lehmann-Willenbrock, Lieselotte Mettler

**Affiliations:** Universitäts-Frauenklinik und Michaelis-Hebammenschule Kiel Michaelisstr. 16 Kiel D-24105 Germany

## Abstract

The operative methods of total uterine mucosal ablation (TUMA) as well as new abdominal and vaginal hysterectomy techniques are described. Classic intrafascial serrated edged macro-morcellator (SEMM) hysterectomy (CISH) by pelviscopy or laparotomy and intrafascial vaginal hysterectomy (IVH) are techniques that allow the nerve and the blood supply of the pelvic floor to remain intact, mainly because only the ascending branches of the uterine arteries are ligated. TUMA avoids the removal of the uterus altogether and is reserved for hypermenorrhea or menorrhagia without major enlargement of the uterus. Both CISH and IVH reduce the physical trauma of hysterectomy considerably and have the advantages of the supravaginal technique. Prophylaxis against cervical stump carcinoma is assured by coring out the cervix with the SEMM. In patients in whom both procedures are possible, IVH is preferred because it combines the minimal trauma and short operative time of vaginal hysterectomy. The decreased diameter of the cervix after coring out greatly simplifies this type of vaginal hysterectomy, the technique that has always been favored because of its short operative times and minimal trauma.


*Diagnostic and Therapeutic Endoscopy*, 1995, Vol. 2, pp. 61–70
Reprints available directly from the publisher
Photocopying permitted by license only

© 1995 Harwood Academic Publishers GmbH
Printed in Singapore

### Laparoscopic and Other Intrafascial Hysterectomy Techniques or Mucosal Ablation—A Choice for Maximum Organ Conservation

KURT SEMM, ENRIQUE LEHMANN-WILLENBROCK and LIESELOTTE METTLER

*Universitäts-Frauenklinik und Michaelis-Hebammenschule Kiel, Kiel, Germany*

*(Received December 31, 1994; in final form May 18, 1995)*

The operative methods of total uterine mucosal ablation (TUMA) as well as new abdominal and vaginal hysterectomy techniques are described. Classic intrafascial serrated edged macro-morcellator (SEMM) hysterectomy (CISH) by pelviscopy or laparotomy and intrafascial vaginal hysterectomy (IVH) are techniques that allow the nerve and the blood supply of the pelvic floor to remain intact, mainly because only the ascending branches of the uterine arteries are ligated. TUMA avoids the removal of the uterus altogether and is reserved for hypermenorrhea or menorrhagia without major enlargement of the uterus. Both CISH and IVH reduce the physical trauma of hysterectomy considerably and have the advantages of the supravaginal technique. Prophylaxis against cervical stump carcinoma is assured by coring out the cervix with the SEMM. In patients in whom both procedures are possible, IVH is preferred because it combines the minimal trauma and short operative time of vaginal hysterectomy. The decreased diameter of the cervix after coring out greatly simplifies this type of vaginal hysterectomy, the technique that has always been favored because of its short operative times and minimal trauma.

**KEY WORDS:** Leiomyoma, fibroid, hysterectomy, laparoscopy, hypermenorrhea, menorrhagia, pelvic floor, pelviscopy

### INTRODUCTION

Minimal invasive surgery follows the principle that only diseased tissue should be removed. When hysterectomy is considered, it is usually only the corpus that is affected i.e., fibromyomata or adenomyosis. Why is then the uterine cervix almost always removed and an "overtreatment" performed.

To understand this, a historical review is helpful. The hysterectomy techniques that are being used today have been developed over many centuries: Soranos from Ephesos described the extirpation of a prolapsed, gangrenous uterus in the first century A.D (1). The first scientifically founded report of a total hysterectomy for cancer was given by Wilhelm Alexander Freund from Breslau in 1878 (2). Later on, however, supravaginal hysterectomy became the method of choice for benign indications, because of its lower complication rates and in particular its lower mortality (3).

Only after about 1963 did total extirpation became the standard procedure, primarily because cervical stump carcinoma had been observed in 0.3 to 1.3% of patients after supracervical hysterectomy (4). For the sake of carcinoma prophylaxis, surgeons accepted the higher complication rates, particularly for urological complications. Thus, the total extirpation became and remained the followed standard, despite the introduction of the routine Papanicolaou smear and its great impact on cervical cancer morbidity and mortality. Therefore, how much follow-up with Papanicolaou smears might have influenced the rate of cervical stump carcinoma has never been evaluated, as this was not performed in the earlier studies (4).

Other disadvantages of the "new dogma" of total hysterectomy soon became apparent. Careful follow-up of patients revealed that total hysterectomy is more detri-

Address for correspondence: Dr. med E. Lehmann-Willenbrock, Universitäts-Frauenklinik und Michaelis-Hebammenschule Kiel, Michaelisstr. 16, D-24105 Kiel, Germany.

mental to their sexual activities than the supracervical method (5). Other authors do not confirm this (6); nevertheless, is is indisputable that total hysterectomy requires ligation of the uterine arteries, amputation of the vagina, and destruction of the nervous uterovaginal plexus along with the cardinal ligaments (Fig. 1). All these structures remain intact when supracervical hysterectomy is performed.

It made sense, therefore, to develop new techniques that combine the minimal invasive approach of the supracervical method with the carcinoma prophylaxis of total hysterectomy (7,8). The procedures described below: classic intrafascial SEMM hysterectomy (CISH) and intrafascial vaginal hysterectomy (IVH) are suitable for this purpose. A new instrument, the serrated edged macro-morcellator (SEMM), efficiently removes the cervical mucosa while the pericervical tissue remains intact (Fig. 2).

When menstrual disorders such as hypermenorrhea and menorrhagia are the only symptoms, even this therapy can sometimes be considered on "overtreatment." If the myometrium is healthy, complete removal of the endometrium is sufficient. Even when the minimal invasive methods of CISH and IVH are used for hysterectomy, the ascending branches of the uterine arteries are ligated, so that the ovarian blood supply may be reduced. This may be the reason why many premenopausal women have climacteric complaints (e.g., hot flushes, etc.) after hysterectomy (9,10).

Since 1987, therefore, gynecologists have been trying increasingly to treat menstrual disorders without myometrial pathological changes not by hysterectomy, but by hysteroscopically guided endometrial ablation (11–15) using high-frequency current (16–19) or laser radiation (20–24). The long duration of the hysteroscopy combined with a destruction of the inner surface of the uterus led to the resorption of considerable amounts of distention media, and thus complications such as lung edema and embolism (25–27). The unpredictable depth of ablation led to injuries (coagulation) of adjacent structures, particularly the intestine (28–30). Aside from these complications, the coagulation effects render a complete histological evaluation of the endometrium impossible. Again, alternative technique were called for. The CISH method inaugurated the development of a total uterus mucosa ablation (TUMA) (31), which removes, analogous to knife conization, the endocervix and endometrium in a cylinder of myometrium with a diameter of 10, 15, 20, or 24 mm. This cylinder is suitable for precise histological evaluation so that the above-mentioned disadvantages of endometrial ablation cease to exist.

### MATERIALS AND METHODS

We included only patients with benign uterine disease requiring hysterectomy or those with therapy resistant menstrual disorders requesting mucosal ablation. To plan the adequate procedure, we performed a bimanual vaginal examination and wet preparation to rule out a vaginal infection. In patients with menorrhagia, dilatation

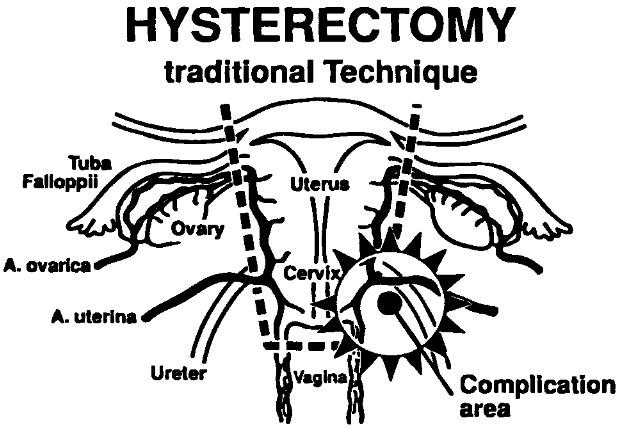

**Figure 1** Scheme for total hysterectomy (e.g., laparoscopic assisted vaginal hysterectomy) with extrafascial extirpation of the uterus and amputation of the vagina.

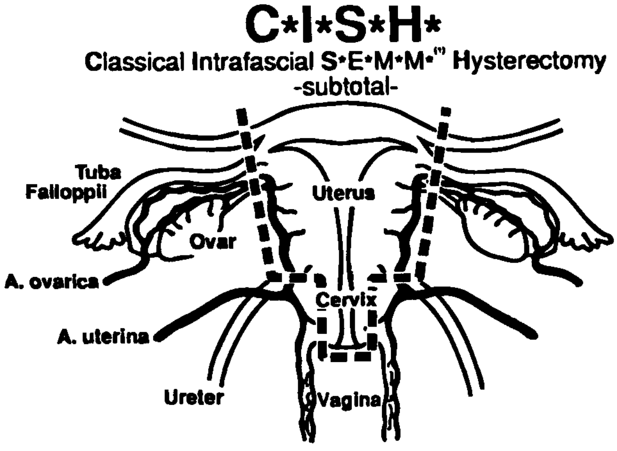

**Figure 2** Scheme for CISH: Intrafascial hysterectomy with coring out of the cervix with a calibrated uterine resection tool (CURT).

and curettage was performed first to exclude endometrial carcinoma. We considered CISH, TUMA, or IVH only if Papanicolaou smear and colposcopic findings were normal; otherwise, a cervical carcinoma had to be ruled out. All benign diseases of the uterus that could not be treated conservatively were considered as an indication for the procedures. Vaginosonography served to rule out malignancy and to take the measurements (see below) required to choose the adequate size of the calibrated uterine resection tool (CURT), i.e., 10, 15, 20, or 24 mm outer diameter.

### Operative Methods

The operative steps for CISH by pelviscopy, IVH, and TUMA may be seen in Figures 3 to 5. For all procedures, a CURT is needed as shown in Fig. 6. Even at pelviscopy, large fibroids may push the CURT off the central track; however, this does not happen within the cervix, which is where it matters. CISH by laparotomy is similar to that by pelviscopy, except that 1) the approach is through a laparotomy incision; 2) before amputation of the uterus, sutures are applied to secure the ascending branches of the uterine arteries; and 3) the cervix is cored out only after the supracervical amputation of the uterus and not before, because the size of the uterus often makes a coring out of the total uterus rather difficult.

In all procedures, the hemostaser/erystop (made by WISAP) was used. This is a steel cylinder that is inserted into the cervix (Fig. 4) or the uterus (Fig. 5), then heated to 120°C by low-voltage direct current and rotated slowly for about 2 minutes, until any bleeding from the cored cervix has stopped. In some patients, we see ectropion such that its diameter exceeds the diameter of the cervix at its narrowest point. In these patients, a conization is performed as part of the procedures (Fig. 7). In most patients this can be avoided if a large enough CURT is chosen. Fig. 8 shows how the CURT functions.

The specimens retrieved by the CISH, TUMA, and IVH procedures are seen in Figures 9, 10, and 11, respectively.

When we chose between the various procedures (TUMA, CISH by pelviscopy or laparotomy, or IVH), the criteria in Table 1 were applied. IVH was performed under pelviscopic control if intra-abdominal pathological findings were was suspected. Applying the criteria of

1) maximum organ conservation,
2) minimal operative time, and
3) short patient recovery, we established an order of preference: TUMA > IVH > CISH by pelviscopy > CISH by laparotomy.

### Patient Follow-Up

We analyzed the first 253 CISH patients between 1991 and 1993, taking into account age, parity, indication for surgery, duration and blood loss of the operation, uterine weight, postoperative complications, and length of stay in hospital. Patients underwent postoperative vaginal examinations 4 to 6 weeks after surgery. In the present study we evaluated the IVH or TUMA patients less extensively, because these procedures were only started in 1993 and there were too few patients to give meaningful numbers to evaluate.

### RESULTS

Among the first 253 CISH patients, nulliparous women were overrepresented in the pelviscopy group (Fig. 12). This may be a result of their smaller uterus size, but the fact that multiparous patients are more suitable for IVH could also be a factor. Age ranged from 40 to 60 years, but there were some younger and some older patients, especially in the pelviscopy groups (Fig. 13). The indication was mostly myoma with bleeding (hypermenorrhea or menorrhagia) or pressure on related organs (Fig. 14).

The intraoperative blood loss was mostly between 200 and 500 ml (Fig. 15); there was no significant difference between pelviscopy and laparotomy. Blood loss at IVH was more difficult to determine precisely. Judging from perioperative hemoglobin and hematocrit changes, we found it to be less than with CISH. Among the first 253 CISH patients, there were 11 postoperative complications: seven hematomas or retention cysts of the cervix (one in the laparotomy group) and four cases of vaginal bleeding (all in the pelviscopy group). Operative time was generally longer by pelviscopy (Fig. 16). In both groups, operative time shortened as experience increased. For the IVH procedure, the operative time was significantly shorter from the beginning, ranging between 20 and 50 minutes. The uterine weight was on average between 100 and 200 g in the pelviscopy group and 200 and 400 g in the laparotomy group (Fig. 17). Most pelviscopy patients stayed in the hospital for 4 to 8 days; most laparotomy patients stayed longer than 8 days (Fig. 18). After IVH, postoperative recovery was also short; patients left the hospital after 4 to 6 days.

Some of our patients returned for a vaginal examination 4 to 6 weeks after TUMA, CISH, or IVH. We saw that the cervix had healed completely at that time, and the former external os had either disappeared or been transformed into a shallow dimple, entirely covered with squa-

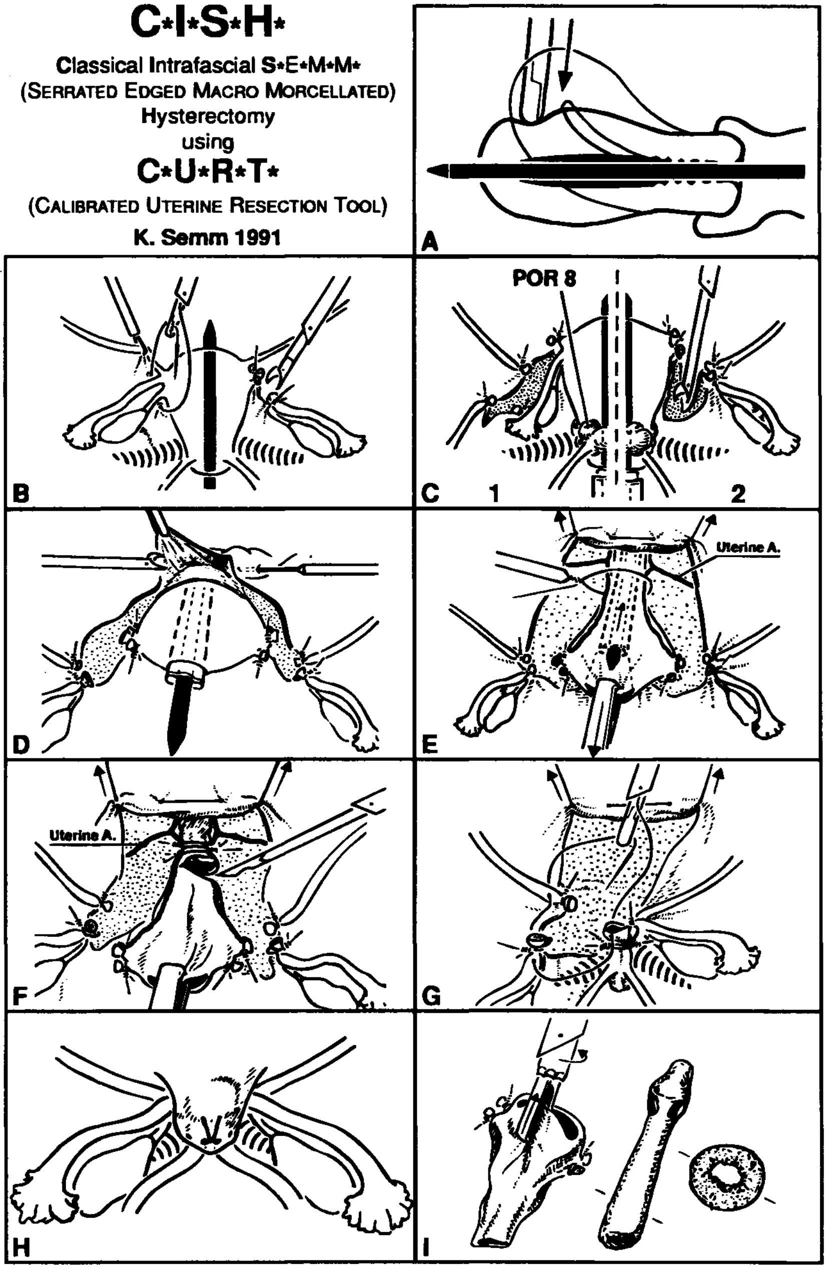

**Figure 3** CISH Technique for laparoscopic hysterectomy without colpotomy. A. Straightening of the uterus by perforation of the fundus uteri. B. Transection of the adnexa in the classical manner between ligatures. C. Opening the parametria, with or without salping-oophorectomy with intra-cervical injection of POR 8. D. Aqua-dissection of the bladder from the cervix, then coring out of the cervix-cavum-fundus cylinder. E. Placing the first SEMM security loop, then total extraction of the CURT with the tissue cylinder, while traction is exerted on the fundus corporis uteri. F. Supracervical transsection of the uterus with scissors or scalpel. G. Fixation of the round ligaments on the cored cervix. H. Peritonealization of the pedicle with the vesico-uterine peritoneum. I. Morcellation of the uterus with the SEMM set; cervix-cavum-fundus-tissue-cylinder; histologic cross-section through the cervix-cylinder.

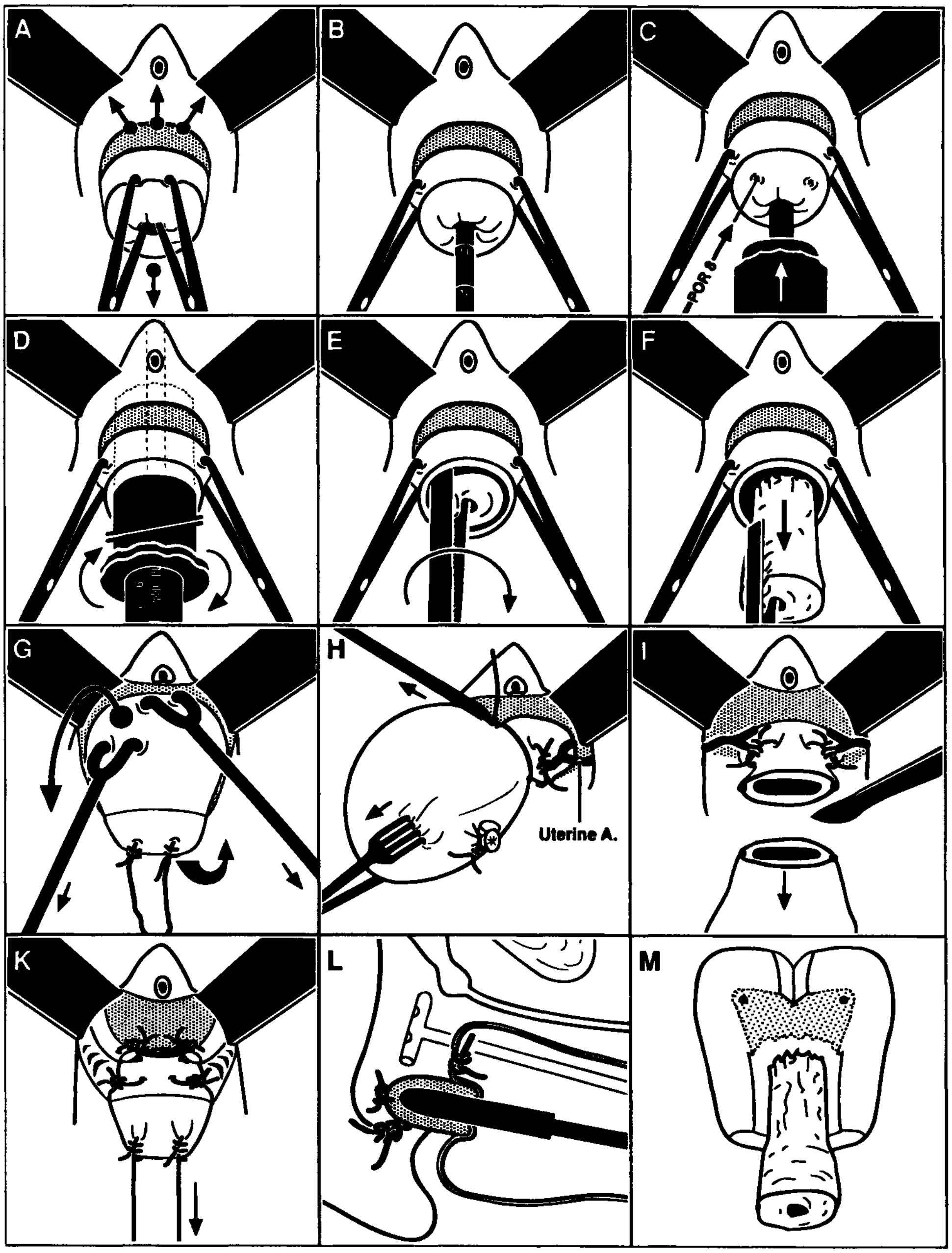

**Figure 4** IVH technique. A. Positioning of two tenacula on the cervix, colpotomy in the anterior plica, and opening the vesicouterine peritoneal fold. B. Transfer of the tenacula deep into the parametria and careful measurement of the uterine sound length. C. Dilatation of the cervical canal, introduction of the perforation rod (blunt end), injection of 2 × 10 ml POR 8 (0.05 IU/ml) into the cervix, then the CURT glides onto the perforation rod. D. Coring out up to two—thirds of the sound length, observing the scale on the CURT. E. After removal of the CURT, twisting the cored cylinder with a grasping forcepts like a myoma in statu nascendi. F. The excised cervix cylinder has been torn off in the cavum uteri by rotation and is now extracted. G. Delivering the fundus uteri through the anterior colpotomy. H. After transection of the adnexas, traction with a roeder loop and ligation of the ascending branches of the uterine artery. I. Supravaginal transection of the uterus with a scalpel. K. Fixation of the round ligaments on the cored cervix, at the same time closing the wound in the cervix. L. Peritonealization of the stumps in continuous suture, then the anterior colpotomy is closed with or without insertion of a drain, and the cervix is coagulated with the WISAP Hemostaser/Erystop. M. Histological specimen.

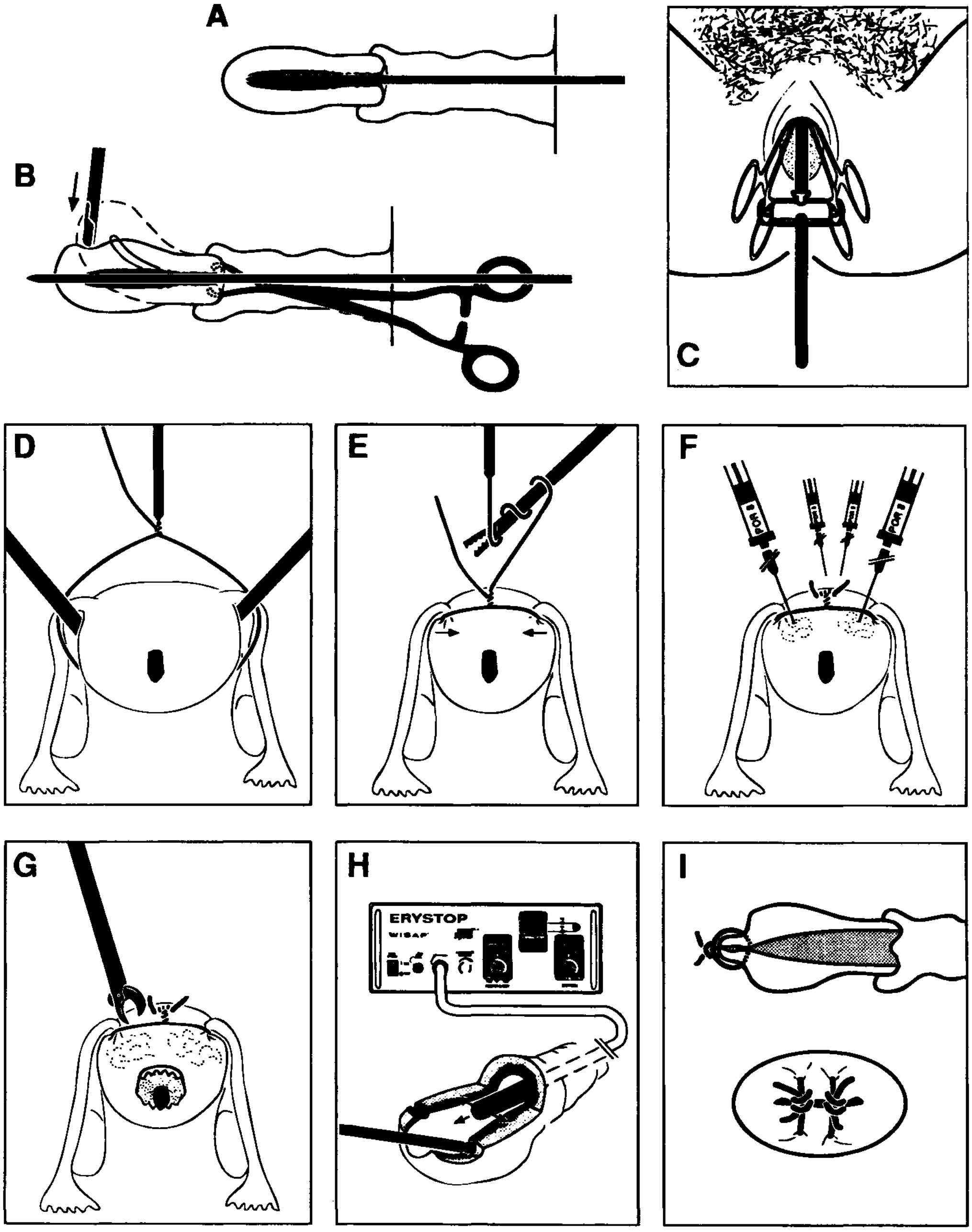

**Figure 5** TUMA with CURT technique. A. After dilation of the cervix, introduction of the 50-cm perforation rod. B. Straightening the uterus with pelviscopic assistance: two tenacula are attached to the pericervical fascia. C. Transvaginal fixation of the perforation rod with the distance holder between the two tenacula. D. With the aid of two biopsy forceps, a SEMM security loop is guided over the fundus uteri and the intramural parts of the tubes. E. After tightening the loop, a security knot is applied. F. Injection of 2 × 10 ml of POR 8 (0.05 IU/ml) into the uterine cervix and the corpus muscle. G. Coring out of the cervix-cavum-fundus cylinder, then transection of the SEMM security loop. H. Extraction of the coring-out device, introduction of the hemostaser, which is then heated to 120°C. The cored muscle is coagulated under pelviscopic hysteroscopic control, after careful inspection of the tissue cylinder, so that well-aimed coagulation of defects can be attained. I. Closure of the defect in the cavum uteri with the CISH needle set: after tying the knot, a second suture with a short needle may be required.

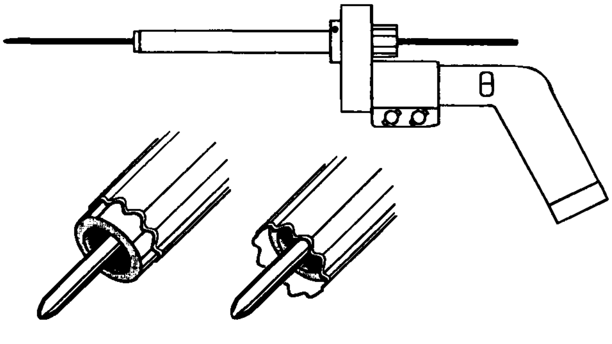

**Figure 6** Morcellating end of the CURT in withdrawn position for insertion into the vagina (with the WISAP motodrive attached) and in cutting position.

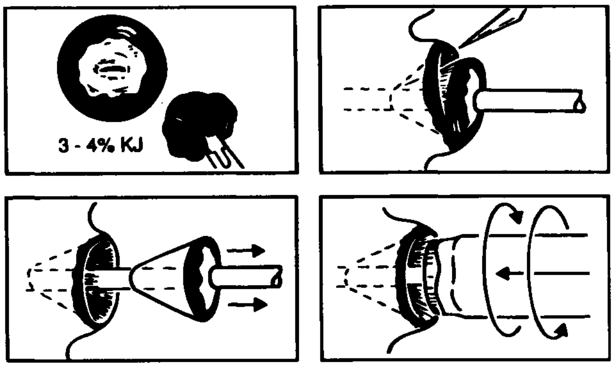

**Figure 7** Conization prior to CISH: Iodine test according to Shiller. B. After bilateral paracervical injection of 2 × 10 ml POR 8 (0.05 IU/ml), the cone is excised with a scalpel. C. The resected cone is removed over the perforation rod: coagulation is effected with the cervix, Hemostaser. D. Later, the cervix is cored with the CURT.

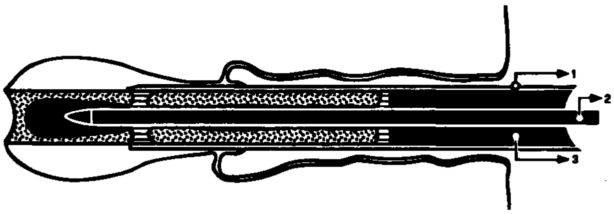

**Figure 8** Extraction of the cored tissue cylinder with the CURT.

mous epithelium. Recovery time after IVH was 1 to 2 weeks.

### DISCUSSION

The indications for TUMA are the same as for endometrial ablation. One difference lies in the fact that, after coring out and coagulation at 120°C, the uterine cavity becomes completely obliterated. This contrasts with endometrial ablation using laser or high-frequency current (e.g., roller-ball), where the canal in its entire length remains open. In summary, TUMA has the following proposed advantages: 1) protection against intrauterine pregnancy; 2) general prophylaxis against cervical and endometrial carcinoma; 3) no cervicitis or cervicomucorrhea; and 4) termination of menstruation as an impediment for the patient. Also, postmenopausal patients receiving hormone replacement therapy would benefit.

For the CISH procedures, operative times were rather long. One reason lies in the fact that most operations were performed by assistants in training. The postoperative stay was also comparatively long. This was due to the setup of the German medical insurance system, whereby no fee was charged for the operation itself, but about $300 (US) for each day the patient stayed in the hospital.

IVH is a classical vaginal hysterectomy through an anterior colpotomy with two new features: posterior colpotomy is completely avoided, and the cervix is cored out with the CURT. The coring out facilitates the removal of the uterus considerably; the cervix becomes thinner, so that it is much easier to pull the fundus uteri through the anterior colpotomy.

Being a vaginal hysterectomy, IVH requires only 20 to 50 minutes operative time. This compares favorably with hysterectomy by laparotomy (160 to 240 minutes) or CISH by pelviscopy or laparoscopic-assisted vaginal hysterectomy (90 to 180 minutes).

The proposed advantages of IVH may be summarized as follows:

1. It is similar to vaginal hysterectomy, a well-established method.
2. The operative time is shorter than with all other hysterectomy techniques.
3. The blood loss is minimal, with or without salpingo-oophorectomy.
4. Pericervical nerves and descending branches of the uterine arteries are not damaged.
5. The wound in the cored cervix heals within 6 weeks.
6. The intact cervical ligaments present an invagination prolapse and a minor prolapse is corrected.
7. Postoperative recovery time is very short

From this list, CISH by pelviscopy still has proposed advantages 4 through 7, whereas CISH by laparotomy maintains advantages 4 through 6.

Only long-term follow-up will show how much cancer and prolapse prevention are improved by CISH and IVH over traditional techniques. Papanicolaou smears have already reduced cervical cancer mortality since the 1960s.

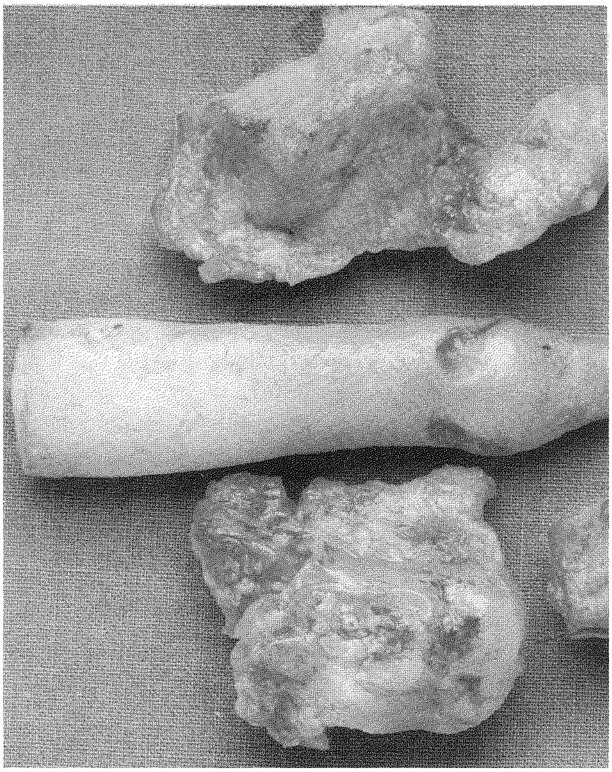

**Figure 9** Histological specimen obtained by pelviscopic CISH.

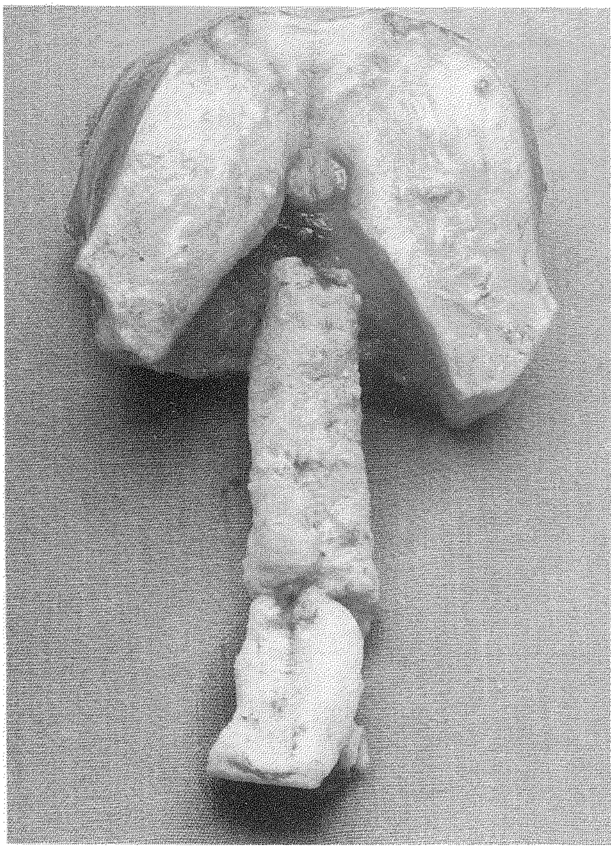

**Figure 10** Histological specimen obtained by IVH.

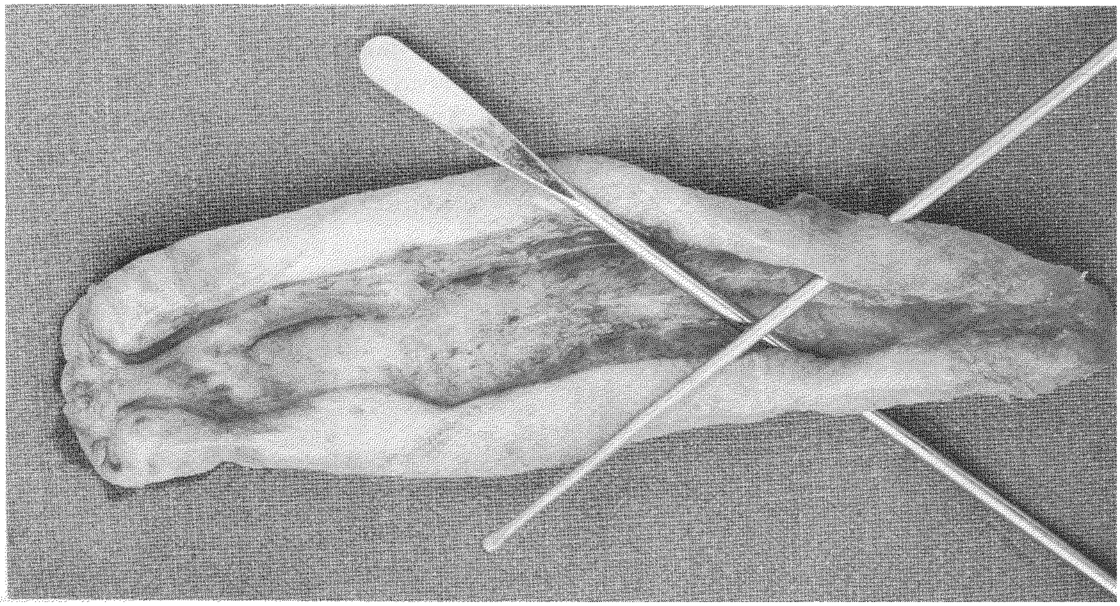

**Figure 11** Histolgical specimen obtained by TUMA

**TABLE 1** Minimal Invasive Surgery on the Uterus—Individualized Therapy According to Indication

| *Indication* | *Recommended Therapy* |
|---|---|
| Sterility and fibroids | Enucleation of the fibroids |
| Hypermenorrhea without paint and without major fibroids | TUMA |
| Pain (e.g., adenomyosis) or fibroids, vagina sufficiently wide | IVH with or without pelviscopic assistance |
| Fibroids, vagina narrow, intra-abdominal adhesions | CISH by pelviscopy |
| Very large fibroids (bigger than 10 cm) | CISH by laparotomy |

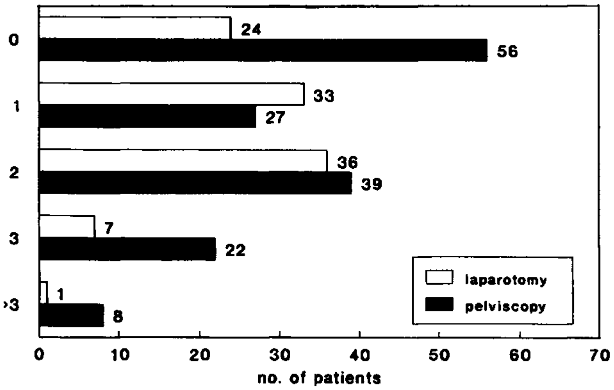

**Figure 12** Parity of patients who underwent CISH.

This, however, should not falsely lead us to omit the coring out step in supracervical hysterectomy, because 1) there are many false-negatives in routine cytology screening, and 2) the treatment of cervical stump carcinoma has worse results than that of cervical carcinoma in the intact uterus.

Considering the advantages of IVH as listed, one may well expect that IVH will be the predominant technique in all cases where it is indicated and possible (Table 1) in the future.

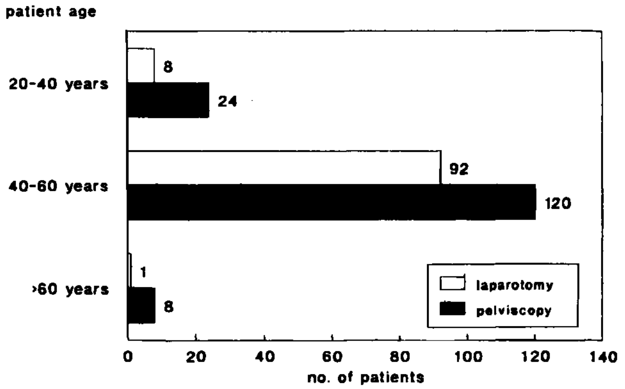

**Figure 13** Age distribution of patients who underwent CISH.

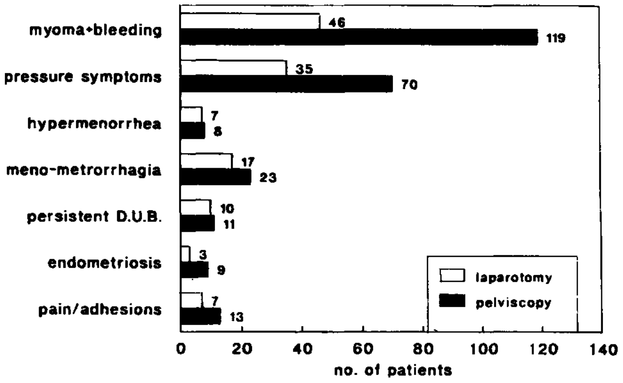

**Figure 14** Indications for CISH.

### REFERENCES

1. Dennerstein L, vanHall E eds. Psychosomatic gynecology. ParkRidge: Parthenon, 1986.
2. Freund WA. Bemerkung zu meiner. Methode der Uterus-Exstirpation. Zentralbl Gynäkol 1878;2:497–500.
3. Ries J. Individuelle Indikation zur Art der Myomoperation. Arch Gynäkol 1961;195:225–227.
4. Tervilä I. Carcinoma of the cervical stump. Acta Obstet Gynecol Scand 1963;42:200. Cited in Kilkku P. Grönroos M. Preoperative electrocoagulation of endocervical mucosa and later carcinoma of the cervical stump. Acta Obstet Gynecol Scand 1982;61: 265–267.
5. Kilkku P. Grönroos M. Hirvonen T. et al. Supravaginal uterine amputation vs. hysterectomy. Effects on libido and orgasm. Acta Obstet Gynecol Scand 1983;62:147–152.
6. Sievers S, Köhler CO. Die vaginale Uterusexstiorpation als. Methode der definitiven Schwangerschaftsverhütung. Geburtsh Frauenheilk 1977; 37:115-123
7. Semm K. Hysterektomie per laparotomiam oder per pelviskopiam. Ein neuer Weg ohne Kolpotomie durch C*A*S*H*. Geburtsh Frauenheilk 1991;51:996–1003.
8. Semm K. Intrafasciale vaginale Hysterektomie (IVH) mit und ohne pelviskopische Assistenz. Geburtsh Frauenheilk 1993;53:873–878.
9. Werth R. Über Ausfallserscheinungen nach abdominaler Myomotomie mit Zurückbelassung der Ovarian. Verh Dtsch Ges Gynäkol Berlin, 1899:140–147.
10. Lehmann-Willenbrock E, Riedel H-H. Ist die Uterusexstirpation eine akzeptable Methode der Schwangerschaftsverhütung? Fertilität 1988;4:179–186.
11. Brun G, Roussilhes R, Saurel J. Endometrial resection for metrorrhagia: 45 cases. J Gynecol Obstet Biol Reprod Paris 1991;20:532–537.
12. Cameron IT. Dysfunctional uterine bleeding. Baillieres Clin Obstet Gynecol 1989;3:315–327.
13. Donnez J, Nisolle M. Hysteroscopic surgery. Curr Opin Obstet Gynecol 1992;4:439–446.
14. Gillespie A. Endometrial ablation: a conservative alternative to hysterectomy for menorrhagia. Med J Aust 1991;154:791–792.

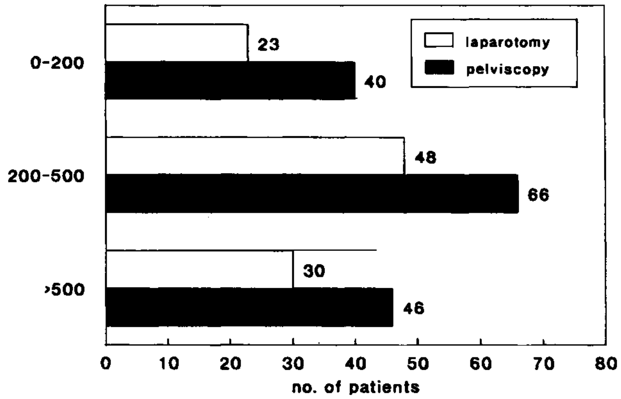

**Figure 15** Intraoperative blood loss for CISH.

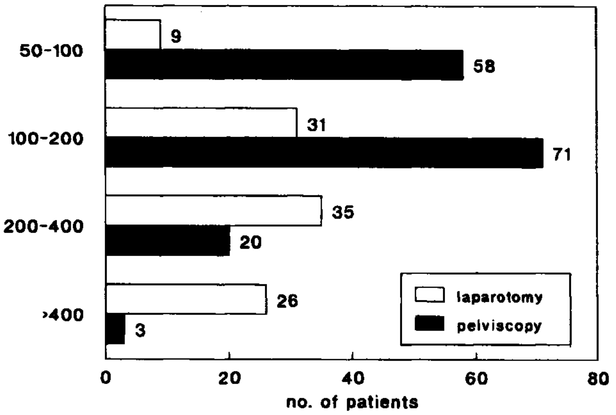

**Figure 17** CISH uterus weight.

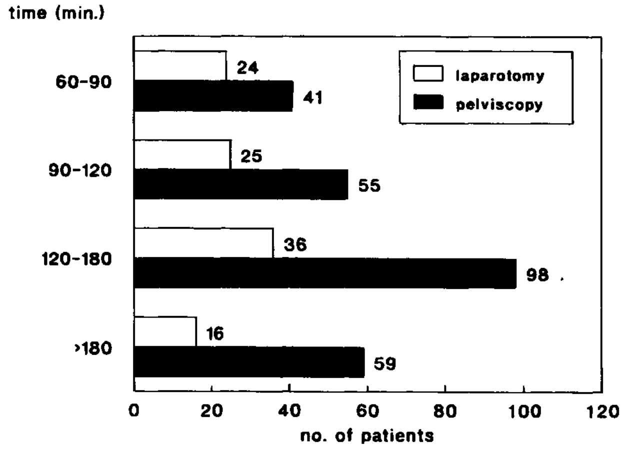

**Figure 16** Operative time for CISH.

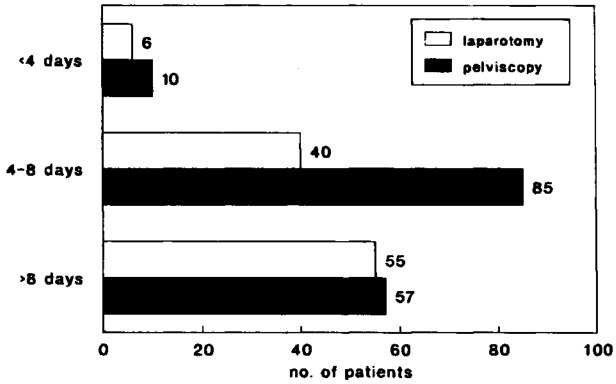

**Figure 18** Hospital stay for CISH.

15. Grainger DA, DeCherney AH. Hysteroscopic management of uterine bleeding. Baillieres Clin Obstet Gynecol 1989;3:403–414.
16. Indman PD, Soderstrom RM. Depth of endometrial coagulation with the urologic resectoscope. J Reprod Med 1990;35:633–635.
17. Lefler HT, Jr, Sullivan GH, Hulka JF. Modified endometrial ablation: electrocoagulation with vasopressin and suction curettage preparation. Obstet Gynecol 1991;77:949–953.
18. Phipps JH, Lewis BV, Roberts T, et al. Treatment of functional menorrhagia by radiofrequency-induced thermal endometrial ablation. Lancet 1990;335:374–376.
19. Prior MV, Phipps JH, Roberts T, et al. Treatment of menorrhagia by radiofrequency heating. Int J Hyperthermia 1991;7:213–220.
20. Bent AE, Ostergard DR. Endometrial ablation with the neodymium-YAG-laser. Obstet Gynecol 1990;75:923–925.
21. Corson SL. Use of the YAG laser in laparoscopic gynecologic procedures. Obstet Gynecol Clin North Am 1992;18:619–639.
22. Goldfarb HA. A review of 35 endometrial ablations using the neodym-YAG laser for recurrent menometrorrhagia. Obstet Gynecol 1990;76:833–835.
23. Lomano J. Endometrial ablation for the treatment of menorrhagia: a comparison of patients with normal, enlarged, and fibroid uteri. Laser Surg Med 1991;11:8–12.
24. Rosenberg C, Tadir Y, Braslavsky D, et al. Endometrial laser ablation in rabbits: a comparative study of three laser types. Laser Surg Med 1990;10:66–73.
25. Hasham F, Garry R, Kokri MS, et al. Fluid absorption during laser ablation of the endometrium in the treatment of menorrhagia. Br J Anaesth 1992;68:151–154.
26. Morrison LM, Davis J, Sumner D. Absorption of irrigating fluid during laser photocoagulation of the endometrium in the treatment of menorrhagia. Br J Obstet Gynecol 1989;96:346–352.
27. Osborne GA, Rudkin GE, Moran P. Fluid uptake in laser endometrial ablation. Anaesth Intensive Care 1991;19:217–219.
28. Baggish MS, Daniell JF. Catastrophic injury secondary to the use of coaxial gas-cooled fibers and artificial sapphire tips for intrauterine surgery: a report of five cases. Laser Surg Med 1989;9:581–584.
29. Clark IW. Necrotizing granulomatous inflammation of the uterine body following diathermy ablation of the endometrium. Pathology 1992;24:32–33.
30. Kivnick SM, Kanter H. Bowel injury from rollerball ablation of the endometrium. Obstet Gynecol 1992;79:833–835.
31. Semm K. Totale Uterus Mucosa Ablatio (TUMA)—C.U.R.T. anstelle Endometrium-Ablation. Geburtsh Frauenheilk 1992; 52:773–777.

